# Lamina Associated Domains and Gene Regulation in Development and Cancer

**DOI:** 10.3390/cells8030271

**Published:** 2019-03-21

**Authors:** Silke J. A. Lochs, Samy Kefalopoulou, Jop Kind

**Affiliations:** Oncode Institute, Hubrecht Institute–KNAW and University Medical Center Utrecht, 3584 Utrecht, The Netherlands; s.lochs@hubrecht.eu (S.J.A.L.); s.kefalopoulou@hubrecht.eu (S.K.)

**Keywords:** nuclear lamina, lamina associated domain, gene regulation, heterochromatin, lamin, DNA methylation, development, cancer, senescence

## Abstract

The nuclear lamina (NL) is a thin meshwork of filaments that lines the inner nuclear membrane, thereby providing a platform for chromatin binding and supporting genome organization. Genomic regions contacting the NL are lamina associated domains (LADs), which contain thousands of genes that are lowly transcribed, and enriched for repressive histone modifications. LADs are dynamic structures that shift spatial positioning in accordance with cell-type specific gene expression changes during differentiation and development. Furthermore, recent studies have linked the disruption of LADs and alterations in the epigenome with the onset of diseases such as cancer. Here we focus on the role of LADs and the NL in gene regulation during development and cancer.

## 1. Introduction

The genome holds the same information for all cells of an organism, but still gives rise to the diverse landscape of cell types that make up all the different tissues. This complexity is driven by differential genome regulation and epigenetic processes, which create a permissive or restrictive environment for gene expression. Spatial genome organization and sub-nuclear compartmentalization play a crucial role in establishing and maintaining these transcriptional environments. One of these sub-nuclear compartments is the nuclear lamina (NL), a thin meshwork of V-type filaments lining the inner nuclear membrane (INM) [1,2]. The meshwork consists mainly of lamin proteins, classified as A- or B-type according to their characteristics, and in mammals are encoded by the *LMNA* (producing both Lamin A and Lamin C) or the *LMNB1* and *LMNB2* genes [2,3,4]. All mammalian cells consistently express one of the B-type lamins, while the levels of A-type lamins change during cell differentiation [5].

Genomic regions contacting the NL are defined as lamina associated domains (LADs) and were first identified using the DamID technique, which maps protein-DNA interactions in a genome-wide manner [6,7,8,9,10]. LADs have been identified in multiple cell types and a diverse range of organisms, such as human fibroblasts, mouse ES cells (mESCs), *Drosophila melanogaster* (common fruit or vinegar fly) and *Caenorhabditis elegans* (a variety of roundworm of the phylum *Nematoda*), indicating that this spatial chromatin organization is conserved throughout evolution [6,7,11,12,13]. LADs are generally gene-poor, have low levels of transcription and are enriched in repressive histone post-translational modifications (PTMs) such as H3K9me2/3 and H3K27me3 [6,14,15]. 

LADs are described as dynamic domains that change spatial positioning in accordance with cell-type specific gene expression activities, suggestive of their involvement in regulating lineage specification [11,16]. Collectively, LADs encompass up to 40% of the genome across different cell types [6,11]. Interestingly, in single cells only approximately one third of the LADs are associated with the NL in a given cell of an isogenic cancer cell line [10]. Changes in the spatial positioning of LADs occurs after mitosis, and a fraction of the NL-detached LADs can be found at the nucleolar periphery, presumably forming nucleolus associated domains (NADs) of heterochromatin with highly similar characteristics to LADs [15,17,18]. LADs are large domains, with a median size of 0.5 Mb [6,11], and larger LADs as well as LAD-dense chromosomes are more frequently in contact with the NL between single cells, suggesting that NL-association is driven by multivalent interactions [10]. A model whereby NL-association is mediated via multivalent interactions is further supported by long stretches of continuous NL-contacts in individual cells [10]. Whether heterogeneity in LAD-organization and multivalent interactions are common phenomena of most cell types, or a specific feature of cancer cells requires further investigation. In this review, we will discuss the function of LADs in genome regulation, development and cancer.

## 2. Transcriptional Repression at the NL

Upon differentiation, multiple genes display movement to or from the NL, concomitantly with changes in chromatin signatures and gene expression [11,19]. These observations point to the NL as a repressive environment, in which genes are either isolated from transcriptional activators or silenced by repressors located at the NL. Over the last decade, there has been a substantial effort to specify a direct role for the NL compartment in gene regulation, for example by an artificial tethering of genomic reporter loci to the NL. However, these approaches have yielded mixed results with regards to transcriptional outcomes [20,21,22]. Tethering of a hygromycin-LacO array to the NL via a LacI-Emerin fusion protein in mouse NIH3T3 fibroblasts results in the decreased expression of the hygromycin gene, which is accompanied by a decrease in histone H4 acetylation [20]. Conversely, a study using a similar LacO system with a LacI-Lamin B1 fusion in human U2OS cells, demonstrated that there is no change in transcription of the reporter upon localization to the NL [22]. The differences between these and multiple other tethering approaches may relate to intrinsic differences in promoter sensitivity to embedding within the repressive LAD chromatin environment [23,24]. In support of this notion, a recent genome-wide study of autonomous promoter activity has shown that LAD promoters indeed display differential sensitivity towards the local chromatin features [25]. This study employed the random integration of LAD promoters into the genome to determine the effect of chromatin context on promoter activity, and identified a class of ‘escaper’ promoters that are relatively insensitive to the LAD chromatin environment [25]. The ability of these elements to escape their repressive surroundings does not directly correlate with promoter strength, but likely depends on inherent DNA sequence features. The discrepancy between transcriptional outcomes of NL-tethering experiments might thus be related to the insensitivity to repression by LAD chromatin of the promoters on some of the reporter arrays. Comparative analysis of the DNA sequence of the ‘escaper’ promoters and the promoters used in NL-tethering studies could provide more insight into this notion. Besides the class of ‘escaper’ promoters, many LAD promoters that are repressed in their native chromatin context become transcriptionally active when they are ectopically expressed from a reporter vector [25]. This change in transcriptional output shows that either LAD chromatin features, or the NL compartment, are responsible for the repressed state of these promoters. Overall, these observations further establish the NL and LAD chromatin as transcriptionally repressive nuclear compartments.

## 3. LAD Organization and Chromatin Features

It is currently still unclear how the spatial organization of heterochromatin at the NL is established, since repressed chromatin could be segregated by active tethering to the nuclear periphery or as a consequence of passive exclusion from the nuclear interior. Recent studies on the heterochromatin protein HP1 show that heterochromatin domain formation is partly mediated by phase separation, illustrating a model in which liquid droplets of HP1 are formed upon DNA binding and subsequent merging of these droplets leads to chromatin compaction [26,27]. Liquid droplet formation could also influence the compaction of heterochromatin at the NL, as a peptide derived from the NL-protein Lamin B Receptor (LBR) decreases phase separation of HP1 in vitro [27]. Potentially, the mechanisms of active tethering, passive exclusion from the interior and phase separation could act in a non-mutually exclusive manner.

Several studies have implicated chromatin features as the main driver for peripheral organization of genomic regions, as forced decondensation of LAD chromatin causes dissociation from the NL and relocation to the nuclear interior [15,28]. Moreover, changes in histone PTMs strongly correlate with dissociation from the NL, as H3K9me2 marks are decreased and H4 acetylation is enriched in dissociated LAD chromatin [15,28]. The histone modifiers G9A, which establishes H3K9me2, and HDAC3, a histone deacetylase, have been shown to interact with the NL and modify LAD chromatin [29,30,31,32]. Indeed, knock-down of G9A causes LADs to partially dissociate from the NL, showing that H3K9 methylation assists the peripheral localization of these domains [15,32]. Interestingly, a study in *C. elegans* identified a new NL protein, CEC-4, which functions as a direct link between H3K9me2/3 marked chromatin and the NL [33]. CEC-4 is exclusively located at the NL and contains a chromodomain, which binds to di- and trimethylated H3K9, making CEC-4 an anchoring point for LADs. Knock-down of CEC-4 caused the release of a transgene array from the NL, without any change in transcriptional levels. This observation supports the notion that chromatin features serve as an active force behind peripheral localization, independent from transcriptional status. Indeed, the detachment of genes from the NL does not depend on their transcriptional activation, as examples in mESCs and human lung carcinoma cells show that many genes relocating to the nuclear interior upon changes in histone PTMs remain inactive [15,28]. Furthermore, the lack of transcriptional activation upon dissociation from the NL shows that the repressive environment of LAD chromatin is not the only cause for the inactive state of these genes, and indicates that additional mechanisms are involved.

So far, no homolog of CEC-4 has been identified in mammals, suggesting that multiple redundant proteins have evolved to tether chromatin to the NL. LBR is a strong candidate for this, as it is located at the NL and can bind H4K20me2 via its Tudor domain [34]. Furthermore, LBR directly interacts with HP1, which binds H3K9me2/3 marks [35,36]. Interestingly, rod photoreceptor cells of nocturnal mammals that lack expression of both LBR and Lamin A/C, exhibit an inverse chromatin organization [37]. In these cells, the compacted heterochromatin is present in the nuclear interior, while the more open chromatin localizes to the NL. CRISPR-Cas9 knock-outs of both LBR and Lamin A/C in other cell types, such as liver or spleen cells, causes the same inverted chromatin phenotype, underlining the role of both of these proteins in tethering of chromatin to the NL [37]. The proline-rich PRR14 protein could be another factor in NL-chromatin association in mammals, since it has a HP1 binding motif and is located at the NL via association with Lamin A/C [38]. Silencing of PRR14 in HeLa cells causes a partial loss of peripheral H3K9me3, and this phenotype is also observed upon combined knock-out of Lamin A/C and LBR [38]. In the coming years, further research on the LAD and NL proteome might shed more light on the key players in LAD chromatin localization in mammals. 

## 4. Spatial Dynamics of Lineage-Specific Genes in Differentiation

**Figure 1:**
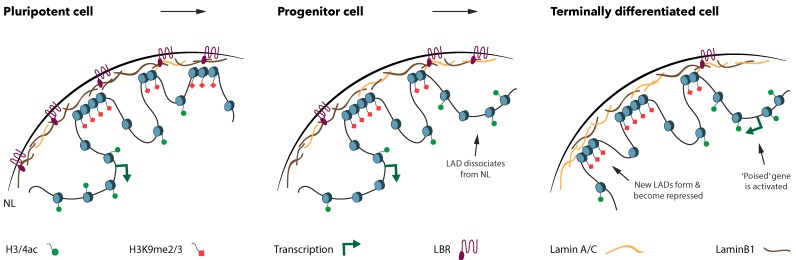
Lamina associated domains (LADs) reorganize during the differentiation from a pluripotent stem cell towards a terminally differentiated cell. Upon cell-fate specification, many spatial genome rearrangements occur. Dissociation of LADs from the nuclear lamina (NL) is concomitant with a decrease in H3K9me2/3 marks and enrichment of histone acetylation. Developmental genes located in the dissociated LADs are not immediately expressed upon release from the NL, suggesting that they are poised for activation in the terminally differentiated cell type. Conversely, pluripotency genes move from the nuclear interior to the NL upon differentiation, which is correlated with a decrease in transcriptional levels, a decrease in histone acetylation and an enrichment in H3K9me2/3. Moreover, the composition of the NL changes during cell-fate specification, as the Lamin B Receptor (LBR) is downregulated while Lamin A/C levels increase.

During the processes of development and cellular differentiation, many spatial genomic rearrangements take place to specify cell fate in a directional manner, during the conversion of a pluripotent stem cell into a terminally differentiated cell (Figure 1). Potentially, the genome plasticity decreases, and the chromatin and transcriptional status of genes are further demarcated. The movement of genes to or from the NL seems to play an important role in cell fate commitment, as these spatial transitions correlate with lineage-specific changes in gene expression [11,39,40]. Upon mESC differentiation towards the neural lineage, multiple pluripotency genes relocate to the NL in the Neural Progenitor Cell (NPC) stage and maintain this localization upon further differentiation to astrocytes [11]. At the same time, neural genes detach from the NL in the transition from mESC to NPCs, often even preceding their transcriptional activation, which only occurs in the terminally differentiated astrocytes (Figure 1). Comparable relocations of lineage-specific promoters are observed during in vitro myogenesis of myotubes [41], upon in vitro differentiation of pluripotent mouse P19 cells towards cardiomyocytes [39,40], and during in vivo differentiation in multiple tissues of *C. elegans* embryos [19]. 

Histone modifying enzymes that contribute to the peripheral localization of genes are also found to play a crucial role in cell differentiation. For example, G9A promotes the differentiation of hematopoietic stem cells, and G9A-deficient mouse embryos show severe growth retardation and early lethality [42,43]. On the other hand, HDAC3 activity is essential to keep cardiac progenitor cells in a pluripotent state, as a knock-out of HDAC3 was shown to cause increased differentiation towards cardiomyocytes [39]. Furthermore, upon knock-out of the *C. elegans* LAD-anchoring CEC-4 protein, heat-shock induction of muscle differentiation in early embryos is less efficient. These defects in the maintenance of pluripotency and the decreases in differentiation efficiency, caused by mutations in LAD associated proteins, show the importance of maintaining proper nuclear localization of genes during development. The observation that dissociation of genes from the NL often does not cause immediate transcriptional activation, suggests that relocation to the nuclear interior poises these genes for activation. It is therefore tempting to speculate that the NL compartment acts by enforcing transcriptional states via shielding of genes from transcriptional activators, and dissociation of these genes from the NL may allow their timely activation during the next stage of development. 

## 5. The Changes in NL Organization in Development 

Besides the repressive function of LAD chromatin, the NL itself contains many interesting proteins that have the potential to directly regulate developmental genes. Lamins can bind histones in vitro, and Lamin A/C is able to bind both active and inactive chromatin in vivo, providing a platform for chromatin association [44,45]. In *D. melanogaster*, Lamin B1 knock-out strongly decreases viability and causes male sterility [46]. 

Furthermore, lamin filaments are also vital in mammalian development, as Lamin A/C knock-out mice are only viable up to a few weeks after birth due to muscular deficiencies, and Lamin B1 or B2 knock-out mice die directly after birth due to lung, skeletal and neuronal defects [47,48,49]. Moreover, the essential function of lamins seems to depend on their nuclear localization. Mutations causing an inefficient incorporation of Lamin B1 into the NL are lethal due to neuronal defects. In addition, Lamin A/C mutations causing a decrease in the nucleoplasmic fraction of the protein are associated with Hutchinson-Gilford Progeria Syndrome [50,51].

Surprisingly, mESCs with a knock-out or knock-down of all three lamins are viable, and have very limited transcriptional changes compared to the wild type cells [52,53,54]. One of the explanations for this independence on the lamin meshwork could be that mESCs are less reliant on a fixed nuclear organization, since they receive smaller mechanical strains compared to more differentiated cell types, and potentially have a more plastic genome structure. The lamin composition also changes during differentiation, as Lamin A/C is very lowly expressed in mESCs and transcriptional levels of the *LMNA* gene are only elevated upon cell specification [55]. Nevertheless, a recent study of triple lamin knock-out mESCs shows that the three-dimensional chromatin structure is mildly affected by the lack of lamins [56]. Briefly, the genome is globally organized into active A- and inactive B-compartments, and can be further partitioned into Topologically Associated Domains (TADs), which are self-interacting structural domains that segregate the spatial genome [57,58,59,60]. The interactions between TADs alter in strength between triple lamin knock-out and wild type cells [56]. Moreover, constitutive LADs that are located at the NL across cell types, generally become more decondensed but remain at the NL. On the other hand, cell type-specific facultative LADs convert to the A-compartment, and become less strongly associated with the NL [56,61]. The partial transitioning of LADs to the active compartment upon lamin loss could be triggered by the inability to embed LADs within the meshwork of lamin filaments, thereby exposing them to chromatin modifiers. However, the minority of transcriptional changes in triple lamin knock-out mESCs indicates that the partial dissociation of LADs from the NL is not sufficient to activate the genes within these domains, and redundant mechanisms are at play. Further research on the direct interactions between LADs and lamins is necessary to elucidate the potential mechanisms in genome organization and to clarify the role of lamins in transcriptional repression with respect to different cell types.

In addition to lamins, LBR also appears to be associated with developmental processes, as it is usually highly expressed in pluripotent cells and becomes downregulated upon differentiation. Downregulation of LBR is often accompanied by the upregulation of Lamin A/C, which for example occurs in proliferative intestinal crypt cells, keratinocytes and olfactory sensor neurons [37,62]. In the latter, decreased levels of LBR lead to formation of large heterochromatin foci of olfactory receptor genes, which repress all but one olfactory receptor in each neuron [62]. Finally, LBR has an important function in X-chromosome inactivation, as it interacts with the X-inactive specific transcript (Xist) long non-coding RNA, and is required for tethering the X-chromosome to the NL [63]. Xist is the general orchestrator of X-chromosome inactivation, in part by activating HDAC3 on the inactive X. The absence of LBR causes a defect in Xist spreading to the active chromatin regions of the X-chromosome and the subsequent silencing of these regions [63,64]. From the studies discussed above, it is apparent that NL proteins themselves influence cell fate specification, potentially by the tethering of LAD chromatin, or by contributing to the repressive capacities of the NL compartment. 

Although much remains to be explored on the mechanisms and key regulators of LAD chromatin organization, a picture emerges in which LADs and the NL environment play an important role during development, as they are potentially involved in guiding and locking in the transcriptional states of many genes. Tethering of developmental genes to the NL supports both the maintenance of pluripotency, and the efficiency of differentiation processes, thereby playing a crucial role in cell fate specification. The role of LADs and the NL in multiple stages of development is further accentuated by the diversity in diseases that can arise upon mutation or removal of NL proteins, such as laminopathies, senescence and cancer.

## 6. LADs Are Potential Major Players in Cancer

Cancer is a disease where the rules of lineage and cell fate specification are disregarded. The initiation of cancer involves dysregulation of gene expression, which results in the loss of cellular identity, and the acquisition of new traits that are favorable for the different stages of tumorigenesis. Over the past few years, it has become increasingly evident that cancer cells not only harbor genetic aberrancies, but also have widespread epigenomic perturbations, due to defects in their epigenetic homeostasis [65,66]. These epigenomic perturbations have the potential to contribute to cancer progression and heterogeneity. However, the role of LADs in cancer and related diseases has hardly been explored, even though the atypical nuclear morphology of cancer cells hints at a defect in the NL structure [67,68]. It is tempting to speculate that LAD reorganization, similarly to the rearrangements during development, results in changes in gene expression that are critical to the establishment and survival of cancer cells. For instance, reprogramming events such as activation of oncogenes or inactivation of tumor-suppressors could be influenced by relocation of LADs, concomitant with epigenetic alterations that cancer cells often harbor. In the coming section, we will review the studies that have linked LADs to epigenetic changes in cancer and pre-malignant processes, like the onset and evasion of senescence. 

Even though LADs have not been studied in direct relation to cancer, an ample amount of information comes from the characterization of another relevant facet of heterochromatin. Large organized chromatin lysine modifications (LOCKs) are genomic regions rich in H3K9me2, which are highly dynamic throughout the differentiation of mESCs, and show an extensive overlap with LADs [69]. Like LADs, LOCKs have been described to reform during lineage specification and some of the genes they contain exhibit differential expression across distinct lineages [69]. Hence, LADs and LOCKs represent highly similar genomic regions, although annotated and studied in distinct ways. Comparison of the epigenome between pluripotent and differentiated cells showed that large H3K9me3 and H3K27me3 domains of repressive chromatin expand when cells commit to a certain fate, potentially locking down pluripotent genes in order to maintain lineage decisions [70]. Observations that H3K9me3 domains create a barrier for the reprogramming of differentiated cells towards induced pluripotent stem cells (iPSCs), further confirm the role of broad heterochromatic domains in cell fate specification [71]. Interestingly, the expansion of heterochromatin seems to be inverted in epithelial to mesenchymal transition (EMT), a crucial cellular reprogramming step in development, as well as in cancer progression. During TGF-β induced EMT, LOCKs have been found to lose their abundance of H3K9me2 and gain transcriptional activity, which is revealed by an enrichment of H3K4me3 at regions with high G/C content and H3K36me3 at LOCK boundaries [72]. In this study, the enrichment of H3K36me3 at LOCK boundaries was concomitant with elevated gene expression at those regions, including genes such as *UNC5B*, *EPHA8* and *LSD1*. Gene ontology analysis of the whole set of genes gaining expression and H3K36me3 enrichment showed possible roles in promoting cell migration, remodeling of the cytoskeleton, and many more EMT-related processes. Thus, these genome-wide chromatin changes seem to be setting the stage for a new transcriptional program, by enabling the release and activation of genes involved in EMT. Collectively, these findings indicate that LAD chromatin can play divergent roles in cell fate decisions that underlie not only normal development but also cancer.

## 7. LADs Are Linked to Loss of DNA Methylation in Cancer

Most of the evidence directly linking LADs to cancer comes from research on DNA cytosine methylation (5mC), an epigenetic mark that has been extensively characterized. DNA methylation is generally considered a repressive mark, with fundamental roles in mammalian development and disease [73,74,75]. It is prominent on gene promoters, mainly on CpG islands surrounding transcription start sites where it contributes to gene repression [76]. Genome-wide mapping of 5mC has shown that differentiated cell types have lower levels of DNA methylation than ESCs and iPSCs [77,78]. This is manifested in the form of partially methylated domains (PMDs), which seem to act as epigenetic memory during cell fate decisions in differentiation, and can even be retained during re-acquisition of pluripotency [78]. 

Exciting insights about LADs and the cancer methylome came from a number of studies that reported the existence of differential DNA methylation in discrete genomic domains [65,79,80,81]. Two of these studies independently demonstrated that large hypo-methylated genomic domains in human colon cancer have a broad overlap with PMDs, LADs and LOCKs. Strikingly, the boundaries of hypo-methylated domains are often enriched in focal DNA hyper-methylation on promoter CpG islands, which can result in repression of tumor-suppressing genes, such as *NRG1* and *SFRP1* [79]. In conjunction with the fact that LAD boundaries are enriched in CpG islands [6], this observation raises the question as to whether or not cancer-specific hyper-methylation at LAD borders is involved in the repression of genes that are positioned at the NL. More examples of transcriptional reprogramming within PMDs include the genes *B3GNTL1* and *TACSTD2*, which both gain expression and reside in regions that lose DNA methylation in colon tumors [68]. Furthermore, colon tumor samples have higher methylation variability compared to control samples, and a considerable amount of cancer-related genes residing in the hypo-methylated LAD-overlapping regions seem to have highly variable expression [65]. This group consists of genes encoding for matrix metalloproteinases (*MMP3, MMP7, MMP10*) that promote cancer metastasis, and genes involved in inflammatory pathways (*CHI3L1*) and tumor metabolism (*STC1*). The stochasticity in the expression of those genes upon hypo-methylation might provide some cells with a selective advantage and, therefore, aid cancer progression. The observations listed above are further endorsed by findings on the metastatic process of pancreatic ductal adenocarcinoma [82]. Upon comparison of distal metastatic foci to primary tumors, the authors observed a redistribution of several chromatin marks in metastasis, including a widespread decrease of H3K9me2. The same samples exhibited DNA hypo-methylation in domains corresponding to LOCKs, which interestingly seemed to be heterogeneous amongst subclones of the same tumor. This strengthens the hypothesis that reprogramming of the epigenome can contribute to cancer heterogeneity.

**Figure 2:**
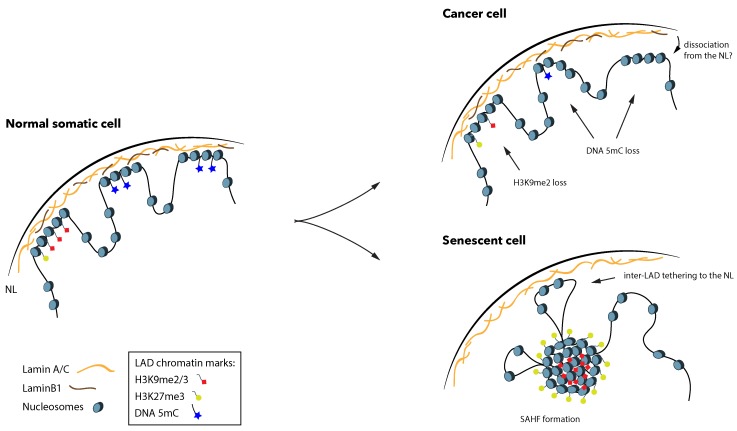
LADs lose DNA methylation abundance in cancer, and undergo dramatic changes in senescence. In a normal somatic cell during interphase, LADs are rich in H3K9me2/3 and H3K27me3 at their periphery. In respect to 5mC, LADs overlap with partially methylated domains (PMDs), where 5mC abundance is lower than in the rest of the genome. Cancer cells display further loss of 5mC from LADs, and in some cases also lose H3K9me2. It is still unknown whether decreased association with the NL accompanies these changes. Senescent cells often lose LaminB1 expression and are characterized by the formation of senescence-associated heterochromatin foci (SAHF), where heterochromatin dissociates from the NL and forms distinct foci in the nuclear interior. Genomic regions that were previously inter-LADs can then make new associations with the NL. Note that, for better visualization, 5mC is omitted from euchromatic non-LAD regions, and is only depicted inside LADs.

The hereby-reviewed studies indicate that LADs in cancer cells are generally depleted of DNA methylation (Figure 2). However, these indications are inconclusive and have not yet allowed for pairwise association and any interpretation of functional links between the epigenome and nuclear organization in cancer. In fact, most of these studies are based on comparisons between independent datasets (e.g., of LOCKs, PMDs and LADs) that represent average measurements, generated with different methods. Thus, they do not validate whether cancer-related DNA hypo-methylation is concomitant with higher or lower association with the NL and gain or loss of certain chromatin marks in the same cell. Since LADs are variable between different cell types and even between single cells of isogenic lines [10,11], it would be of great value to understand DNA methylation changes relatively to LAD dynamics and the accompanying chromatin facets from the same samples. Moreover, it is still unclear how genome-wide differential methylation occurs, and what its implications are in the initiation and progression of cancer. Findings from a study in breast cancer indicated that tumor-suppressor genes contained in DNA hypo-methylated domains are significantly downregulated, due to the accompanying formation of repressive heterochromatin in the hypo-methylated regions [80]. In combination with the observation that loss of DNA methylation provokes stochasticity in gene expression [65], this indicates that widespread DNA hypo-methylation in cancer might not necessarily lead to gene activation. In fact, DNA methylation has variable context-dependent roles, exemplified by the fact that it is commonly found to be abundant on the promoters of lowly expressed genes, but also in the gene bodies of highly expressed genes [81,83]. 

Noteworthy is a recent study shedding light upon the occurrence of genome-wide 5mC depletion [84]. There, the authors demonstrated that loss of DNA methylation in a certain locally defined CpG context of PMDs is shared across developmental lineages and several cancer types. This seems to correlate with the accumulation of cell divisions and the late-replication timing of PMDs, presumably because maintenance of 5mC is inefficient in those regions after each cell cycle. Since PMDs show an extensive overlap with LADs and heterochromatin, this effectively points out that the loss of 5mC from LADs may gradually occur during cellular aging, and loss of DNA methylation in these domains might serve as an “epigenetic clock” for the cell’s age. Thus, LADs and wide-range DNA methylation dynamics are tightly linked, probably representing a physiological process where loss of 5mC initiates early in the cell’s lifespan, intensifies upon ageing and appears as a profound feature of cancer. How gradual DNA methylation loss from heterochromatin can be so ubiquitous in normal development and seemingly able to impute cancer cells with favorable features, is a rather compelling question.

## 8. LADs Are Extensively Redistributed during Cellular Senescence

Another imminent role of LADs relevant to cancer is depicted by the chromatin features they adopt during senescence. Senescence is a pivotal process in the lifespan of a cell, as it seems to be imposing a “decision node” on the path towards tumorigenesis and is a key aspect of embryonic development as well as ageing [85,86,87,88,89,90,91]. Senescent cells undergo an irreversible arrest in cycling and proliferation that eventually leads to cell death [92]. Vice versa, cells that bypass the tumor-suppressing mechanisms of senescence can become pre-malignant and subsequently initiate cancer [93,94,95]. The involvement of chromatin dynamics in senescence has been illustrated by a number of insightful studies over the recent years. Cells that undergo replicative senescence in vitro exhibit the same widespread domains of DNA hypo-methylation that overlap with late-replicating heterochromatin and LADs, as described previously for cancer cells [96]. This seems to be dependent on failure of the DNA methyltransferase DNMT1 to confine to late-replicating regions and restore methylation after DNA replication [84]. DNA hypo-methylation of LADs in senescence is apparently accompanied by matching changes in histone modifications, as senescent cells acquire broad H3K4me3 and H3K27me3 enrichments [97], on the same hypo-methylated LAD-overlapping regions described previously. Hence, these correlative studies indicate that LADs of senescent cells bear epigenetic reprogramming similar to that of cancer cells, an observation that is quite remarkable given that senescence is an obstacle to carcinogenesis. Yet, these findings support the notion that progressive loss of DNA methylation from heterochromatin might indeed have a cumulative role in normal cell fate, instead of being a feature that emerges with the onset of cancer or senescence. In this way, an aged cell that has approached the pre-senescent/pre-cancer state might already harbor the LAD epigenomic landscape observed in senescent and cancer cells. A parallel theory could be that the epigenomic similarities of cancer and senescence are the outcome of retained features carried by cells that go through senescence but manage to escape, regain proliferation and eventually give rise to cancer [96]. This implies that although the epigenomic features of senescent cells should be anti-tumorigenic, they may actually favor cancer initiation when maintained by cells that evade senescence, a notion that provides a rough explanation on the late-life onset of many cancers. The findings described above provide only correlations of the epigenomic features found in LADs between senescent and cancer cells. Accordingly, comprehensive studies in which the LAD organization is profiled in association with epigenomic changes could improve our understanding about these events in senescence and cancer.

One of the hallmarks of senescence, evidently linked to LAD reformation, is the accumulation of senescence-associated heterochromatin foci (SAHF). These are described as the result of a global re-arrangement phenomenon that involves the dissociation of heterochromatin from the NL and relocation to the nuclear interior [98] (Figure 2). SAHF are enriched in H3K9me3 in their center and H3K27me3 on their periphery [99]. Strikingly, oncogene-induced senescence is accompanied by downregulation and redistribution of Lamin B1, which loses associations with H3K9me3-rich domains and gains interactions with H3K27me3-rich domains [100]. This indicates that dysregulation of Lamin B1 might be fostering a pre-SAHF nuclear landscape. Complementing these observations, a Hi-C study revealed that senescent cells not only display striking changes in global nuclear interactions, but also loss of local interactions between heterochromatic regions [101]. They specifically focused on TADs, showing that TADs that lost most local interactions in senescence, defined by loss of their boundary insulation, were also enriched in LADs, A/T content and H3K9me3, overall representing constitutive heterochromatin. These areas correspond to the same previously identified domains that exhibit loss of binding by Lamin B1 [100]. Notably, the authors also reported newly formed senescence-specific LADs that are G/C-rich and are encompassed by TADs that gain boundary insulation during senescence [101]. These findings were further confirmed with the use of DamID [102], which demonstrated that oncogene-induced senescent cells are depleted of constitutive LADs, but gain interactions in inter-LAD domains. Interestingly, the genes that relocate to the NL are not repressed, indicating that in senescence any repressive function of the NL is abrogated. Therefore, the LAD landscape of senescent cells undergoes vast spatial rearrangements, which are highlighted by a loss of heterochromatin-lamina association. Taken together, these observations demonstrate that the global nuclear architecture of senescent cells is rather inverted compared to pre-senescent cells, and this inversion is ultimately revealed by the formation of SAHF.

## 9. Concluding Remarks

The pivotal role of spatial genome organization in normal developmental processes, as well as disease, has become increasingly evident. During cell fate specification, LADs reorganize to release or associate genes with the NL, with variable context-dependent outcomes on cellular fate. The causal links between NL association and transcription are difficult to decipher, since recent evidence shows that NL localization does not exclusively lead to gene repression. Many NL-associated proteins seem to be essential throughout development, further supporting the hypothesis that the NL and three-dimensional genome structure lie at the core of developmental regulatory mechanisms. In ageing and cancer, the LAD chromatin undergoes dramatic changes such as loss of DNA methylation and redistribution of heterochromatin features. The interplay between LADs and DNA methylation dynamics is still poorly understood, even though it seems that broad DNA methylation loss might have a cumulative impact during cellular lifespan. Recent studies in the field of genome organization have brought many insights and, at the same time, raised numerous fascinating questions. We still do not fully understand the cues that stimulate LAD reorganization and the timing of these reorganizations on the developmental trajectories. Likewise, in cancer, the time frame of LAD chromatin reprogramming and nuclear reorganization is still unclear, and it is not yet addressed whether or not these changes can facilitate cancer heterogeneity. In the near future, state-of-the art multiomic sequencing technologies and in vitro development and disease models can elucidate these questions, and bring further knowledge on the significant role of LADs in development, ageing and cancer.
